# Beyond eNOS: Genetic influence in NO pathway affecting drug response

**DOI:** 10.1590/1678-4685-GMB-2022-0157

**Published:** 2022-10-14

**Authors:** Aline Esposito, Cezar Kayzuka Cotta, Riccardo Lacchini

**Affiliations:** 1 Universidade de São Paulo, Departamento de Farmacologia, Ribeirão Preto, São Paulo, SP, Brazil.; 2 Universidade de São Paulo, Departamento de Enfermagem Psiquiátrica e Ciências Humanas, Ribeirão Preto, São Paulo, SP, Brazil.

**Keywords:** Nitric oxide, polymorphisms, drug response

## Abstract

Nitric Oxide (NO) has important biological functions, and its production may be influenced by genetic polymorphisms. Since NO mediates the drug response, the same genetic polymorphism that alter NO levels may also impact drug therapy. The vast majority of studies in the literature that assess the genetic influence on NO-related drug response focus on *NOS3* (which encodes endothelial nitric oxide synthase), however several other proteins are interconnected in the same pathway and may also impact NO availability and drug response. The aim of this study was to review the literature regarding genetic polymorphisms that influence NO in response to pharmacological agents located in genes other than *NOS3.* Articles were obtained from Pubmed and consisted of 17 manuscripts that assessed polymorphisms of the following targets: Arginases 1 and 2 (*ARG1* and *ARG2*), dimethylarginine dimethylaminohydrolases 1 and 2 (*DDAH1* and *DDAH2*), and vascular endothelial growth factor (*VEGF).* Here we analyze the main results of these articles, which show promising evidences that may suggest that the NO-driven pharmacological response is affected by more than the eNOS gene. The search for genetic markers may result in better understanding of the variability of drug response and turn pharmacotherapy involving NO safer and more effective.

## Introduction

One of the main active molecules produced by endothelial cells is nitric oxide (NO), a small gaseous and lipophilic molecule, which acts in smooth muscle of vessels leading to vasorelaxation. NO is one of the most important molecules that regulate blood pressure and flow (Moncada and Higgs, 1993). 

NO targets soluble guanylate cyclase, which is an enzyme responsible for converting guanosine triphosphate (GTP) into cyclic guanosine monophosphate (cGMP). Increasing levels of cGMP, in turn, activate Protein Kinase G (PKG), which will phosphorylate several targets, resulting in reduced cytoplasmatic calcium and vascular relaxation (Craven and DeRubertis, 1978; Poulos, 2006; Francis et al., 2010). 

Nitric oxide is mainly produced by NO synthases (NOS), which catalyze the conversion of L-Arginine into L-Citruline and NO. There are three types of NOS: neuronal (nNOS, encoded by *NOS1*), inducible (iNOS, encoded by *NOS2*) and endothelial (eNOS, encoded by *NOS3*) (Kiechle and Malinski, 1993; Silva et al., 2011). The nNOS and eNOS enzymes are expressed in different cell types, including neurons and endothelial cells. Both enzymes are calcium-dependent constitutive isoforms, which increase their catalytic velocity in response to increases in calcium, through activation of calmodulin (CaM). On the other hand, iNOS is not constitutive, showing a marked upregulation in response to inflammation (Cinelli et al., 2020).

**Figure 1 -:**
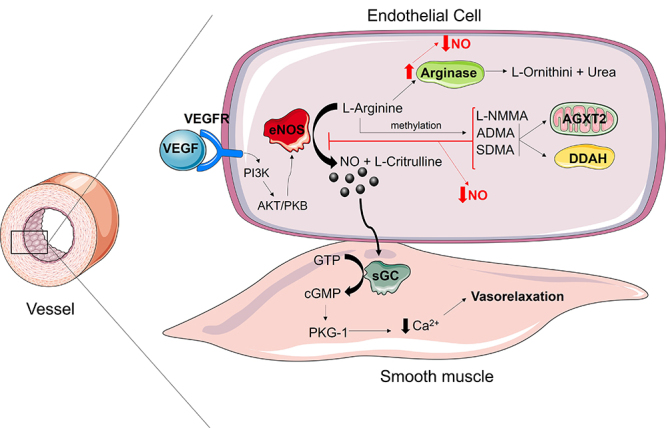
Schematic figure of the NO-cGMP pathway. NO is synthesized by eNOS, diffuses through the membrane and activates sGC, which in turn increases cGMP that leads to vasorelaxation. L-NMMA, ADMA and SDMA compete with L-Arginine and reduce NO synthesis. DDAH1 and 2 and AGXT2 metabolize these methylated forms of L-Arginine. Arginases limit the availability of L-Arginine. VEGF induces the expression of eNOS. NO, Nitric oxide; eNOS, Endothelial nitric oxide synthase; ADMA, Asymmetric Dimethylarginine; SDMA, Symmetric Dimethylarginine; L-NMMA, Monometilarginina Simetrica; DDAH, Dimethylarginine dimethylaminohydrolase; AGXT2, Alanine glyoxylate transaminase-2; sGC, Soluble guanylate cyclase; GTP, Guanosine triphosphate; cGMP, Cyclic guaninosine monophosphate; PKG-1, Proteína quinase-G 1; VEGF, Vascular endothelial growth factor; VEGFR, Vascular endothelial growth factor receptor; PI3K, Phosphoinositide-3-kinase; AKT/PKB, Protein kinase B.

The NO pathway is complex and involves other enzymes upstream or downstream of the NO signal (Figure 1). For instance, Arginase 1 and Arginase 2 are enzymes that compete for the same substrate of NOS and may limit NO production (Caldwell et al., 2018). Besides that, there are methylated forms of L-Arginine that act as NOS inhibitors, such as asymmetrical dimethylarginine (ADMA), symmetrical dimethylarginine (SDMA) and monomethylarginine (L-NMMA) (Schepers et al., 2014). While the production of methylated forms of L-Arginine is very complex, and mainly due to degradation of proteins that had L-arg residues post-translationally modified, the clearance of these molecules is very well identified, being performed by dimethylarginine dimethylaminohydrolases types 1 and 2 (Valkonen et al., 2005), and by alanine-glyoxylate aminotransferase type 2 (Rodionov et al., 2014). Besides that, the Vascular Endothelial Growth Factor (VEGF), Hypoxia Inducible Factor 1 (HIF-1), acetylcholine, mechanical stretch on endothelial cells, and others are able to activate or upregulate NOS (Melincovici et al., 2018). The mentioned proteins and enzymes have genetic polymorphisms that may impact their action by altering their expression, activity, affinity to other ligands, and other consequences. Indeed, polymorphisms in *ARG1, ARG2, DDAH 1, DDAH 2, AGXT 2* and *VEGF* were associated to altered risk for cardiovascular diseases, diabetes mellitus and preeclampsia (Leineweber et al., 2017). Moreover, there is evidence showing the association of polymorphisms in NO pathway genes with altered response to drugs (Cotta Filho et al., 2020), including those that involve NO in their pharmacological mechanism. The most obvious target for genetic association would be *NOS3* gene, which has been largely explored, but other genes that participate in NO pathway will also impact NO availability and may also modulate risk for disease and drug response (Valkonen *et al.*, 2005; Dobrian, 2012).

Here we aimed to review the literature regarding genetic polymorphisms that influence drug response involving NO, but focusing studies that went beyond *NOS3*.

## Literature search

**Figure 2 -:**
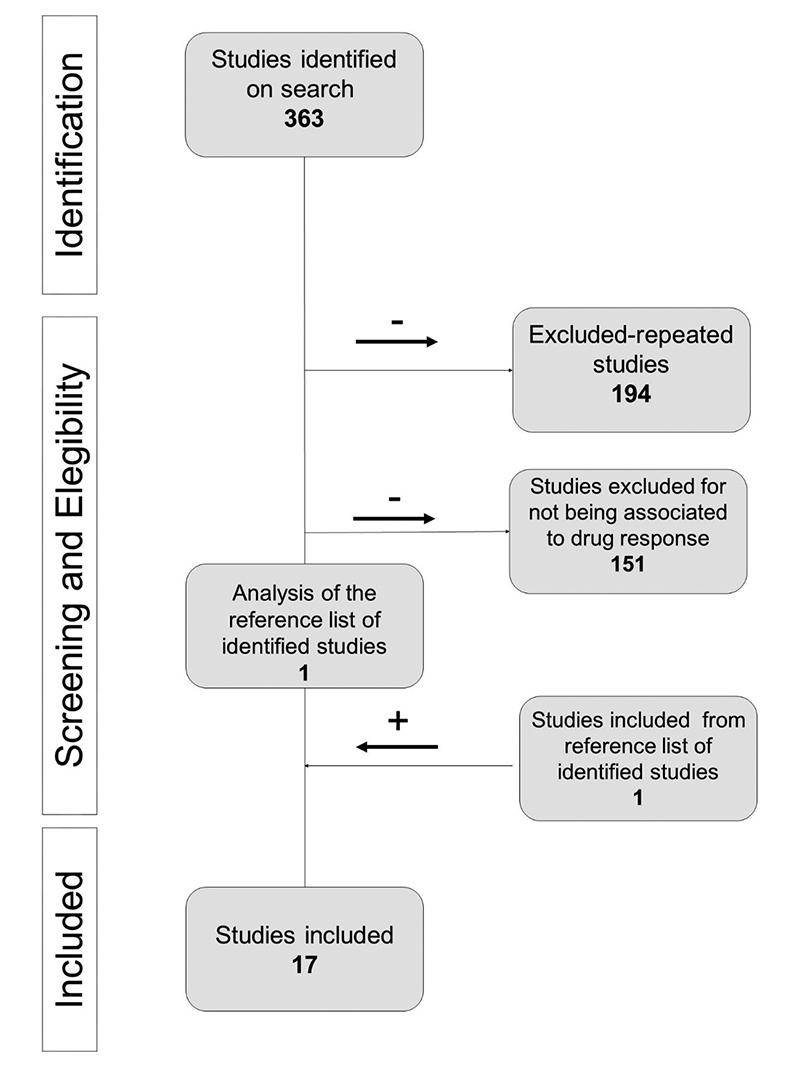
The article selection process. Terms used on PubMed for Search Title/Abstract AND Crossing the terms listed in the method section.

Our search was based on Pubmed using the following search terms: Polymorphisms; Nitric oxide; Arginase 1;ARG1; Arginase 2; ARG2;Alanine glyoxylate transaminase-2; AGXT2; Dimethylarginine dimethylaminohydrolase-1;DDAH1;Dimethylarginine dimethylaminohydrolase-2 ;DDAH2; Asymmetric Dimethylarginine; ADMA;Symmetric Dimethylarginine; SDMA; Endothelial growth factor; VEGF; Soluble guanylate cyclase; sGC. These terms were searched in title and abstracts throughout the database. Figure 2 describes the selection process of articles included here. All articles were original and in English language, including polymorphisms of *ARG1, ARG2, DDAH1, DDAH2* and *VEGF* and its association with drug response. As eNOS was extensively studied in other articles (Silva et al., 2011; Oliveira-Paula et al., 2017; Cozma et al., 2019; Cotta Filho et al., 2020), and given the idea proposed here of emphasizing what lies beyond *NOS3,* we excluded all references focusing only on *NOS3*. Besides, articles that focused on disease risk phenotypes were also excluded, except for targets not explored by pharmacogenetic studies (soluble guanylate cyclase and *AGXT2*). References of the included articles were double-checked to include new studies not identified originally by our Pubmed search. After the selection process, 17 articles were included and explored in this review. 

## Arginase 1 and Arginase 2

Arginase 1 (*ARG1*) and Arginase 2 (*ARG2*) are enzymes that catalyze the hydrolysis of L-arginine into L-ornithine and urea (Caldwell et al., 2018). The two arginase isoenzymes differ by tissue expression, subcellular localization, and immunological reactivity while maintaining 60% homology in protein sequence (Vockley et al., 1996). Since arginases use the same substrate as NOS, they compete and may limit NO synthesis by eNOS and nNOS through microcompartment exhaustion of L-Arginine (Romero et al., 2008). This effect is substantial and may explain the role of arginases in endothelial dysfunction observed in cardiovascular diseases (Romero *et al.*, 2008; Johnson et al., 2015). *ARG1* is located in the long arm of chromosome 6, while *ARG2* is located on the long arm of chromosome 14, and both have polymorphisms with clinical importance.

Genetic influence of ARG1 and ARG2 in asthma treatment responsiveness

The vast majority of pharmacogenetic studies involving *ARG1* and *ARG2* concentrate on response to drugs used in asthma (Table 1 and Table 2). This is due to an increased activity of arginases in asthma pathogenesis, which in turn lead to reduced NO synthesis and obstruction of airways. Additionally, this leads to increased inflammation and remodeling of airways (Maarsingh et al., 2008).

**Table 1-:** Summary of studies that evaluated the association of polymorphisms in *ARG1* with response to drugs.

Polymorphism	Study Population	Disease	Drug	Main Findings	Reference
rs2781659 (A>G) rs2781667 (C>T) rs2246012 (T>A) rs17599586 (C>T)	Brazilian	Erectile dysfunction	Sildenafil	No significant associations	(Lacchini et al., 2018)
rs2781659 (A>G) rs2781667 (C>T) rs2246012 (T>A) rs17599586 (C>T)	Brazilian	Colonoscopy	Propofol	Ni affect on the propofol-induced changes in blood pressure and heart rate	(Oliveira-Paula et al., 2021)
rs2781659 (A>G) rs2781663 (T>A) rs2781665 (A>T) rs2749935 (A>C)	Non-Hispanic white Non-Hispanic black Hispanics Other	Asthma	Bronchodilator (BD) β _2_ agonist Glucocorticoid	rs22781659 significantly associated with BD response	(Litonjua et al., 2008)
rs2781667 (C>T) rs2781668 (C>T) rs17599586 (C>T)	Europeans			T-allele of rs2781667 associated with a significantly decreased response to β _2_ agonist	(Vonk et al., 2010)
rs2781659 (A>G) rs2781663 (T>A) rs2781665 (A>T) rs60389358 (C>T)	Non-Hispanic whites African Americans Hispanics			Haplotype ATAC carriers were more likely to have higher response BD	(Duan et al., 2011)
rs2781659 (A>G)	Caucasian			No significant association	(Scaparrotta et al., 2019)
rs2781667(C>T)	Russians Tatars Bashkirs			No significant associations	(Savelieva et al., 2020)

**Table 2 -:** Summary of studies that evaluated the association of polymorphisms in *ARG2* with response to drugs.

Polymorphism	Study Population	Disease or Procedure	Drug	Main Findings	Reference
rs3742879 (A>G) rs10483801 (C>A)	Brazilian	Erectile dysfunction	Sildenafil	No significant associations	(Lacchini et al., 2018)
rs3742879 (A>G) rs10483801 (C>A)	Brazilian	Colonoscopy	Propofol	AG + GG genotypes for the rs3742879 polymorphism in ARG2 gene and the ARG2 GC haplotype (rs3742879 and rs10483801) show lower increases in nitrite levels and lower decreases in blood pressure after propofol anesthesia	(Oliveira-Paula et al., 2021)
rs2145467 (T>A) rs2295643 (A>T) rs17249437 (T>C) rs17249444 (G>C) rs12896052 (G>A) rs7140310 (T>G) rs3742879 (A>G) rs10483801 (C>A)	Europeans	Asthma	Bronchodilator (BD) β _2_ agonist Glucocorticoid	Allele G of rs7140310 and allele A of rs10483801 increased bronchodilatation response for β _2_ agonist	(Vonk et al., 2010)
rs17249437 (T>C) rs3742879 (A>G) rs7140310 (T>G)	Russians Tatars Bashkirs			TT and GG genotypes of rs17249437 and rs3742879 respectively were associated with a decline in lung function	(Savelieva et al., 2020)
rs10483801 (C>A)	African-Americans	Sickle cell anemia	Hydroxyurea	CA and AA genotype carriers in SNP rs10483801 showed changed level of fetal hemoglobin after treatment	(Ma et al., 2007)
rs2295643 (A>T)	Europeans	Myeloproliferative neoplasms	Hydroxycarbamide	No significant differences	(Angona et al., 2013)

The class of β-agonists is widely used in asthma treatment, with short-term acting β_2_ agonists usually used to promote ailment to the acute symptoms of bronchospasm, while long-term β_2_ are more often used along with inhaled corticosteroids in a chronic treatment. This class exerts therapeutic effects through activation of the β_2_ receptor, which is more expressed in smooth muscle cells of the lower respiratory tract. This results in an increase in cyclic adenosine monophosphate (cAMP) in the cellular milieu, which activates PKA and results in bronchodilation (Tse et al., 2011). 

Genetic polymorphisms of *ARG1* and *ARG2* were associated with the risk to develop asthma (Li et al., 2006; Vonk et al., 2010) and the response to β_2_ agonists (Litonjua et al., 2008). An important study assessing this effect was a panel of 844 SNPs on 111 candidate genes, which reported an association of the rs2781659(A>G) of *ARG1* with bronchodilator effectiveness both in children and adults (Litonjua *et al.*, 2008). It was shown that carriers of variant G allele of the rs2781659 had a diminished response to the drug when compared to the wild type AA. Another study assessed the association of SNPs in *ARG2* with response to β_2_agonists and anticholinergic bronchodilators (Vonk *et al.*, 2010). It was shown that *ARG1* rs2781667(C>T) T carriers had a reduced bronchodilator response to β_2_ agonists, while *ARG2* rs7140310(T>G) and rs10483801 (C>A) variant alleles showed an increased response to the same drugs. No association was reported regarding anticholinergic bronchodilators (Vonk *et al.*, 2010). Interestingly, the pharmacological response to asthma therapy is usually assessed by quantifying the forced expiratory volume in one second (FEV_1_). Inhaled corticoid therapy was more effective in the reduction of FEV_1_ of carriers of *ARG1* rs2781667(C>T) variant T allele when compared to the CC genotype (Vonk *et al.*, 2010). Contrasting previous results, another study reported no association of *ARG1* rs2781659 (A>G) with bronchodilator response (Scaparrotta et al., 2019). 

Further studies explored the *trans* interaction between genetic polymorphisms in the form of genotype interaction (Sy et al., 2012), with interesting results showed in a Chinese population. It was found that the interaction between ARG1 (rs2749935) and the Corticotropin Releasing Hormone Receptor 2 (CRHR2) (rs2190242) polymorphism could alter bronchodilator responsiveness in asthmatics. The results show that those patients classified as high risk (i.e. ARG1 rs2749935 AA or CC genotype with CRHR2 rs2190242 CC genotype) have better bronchodilator responsiveness than low-risk genotype carriers (i.e., ARG1 rs2749935 TA genotype with CRHR2 rs2190242 AA genotype) by generalized multifactor dimensionality reduction. Duan et al. (2011) assessed haplotypes formed by 4 SNP in *ARG1*: s2781659(A>G), rs2781663(T>A), rs2781665(A>T) e rs60389358(C>T). They compared three different haplotypes and showed that the variant haplotypes GATC and GATT responded worse than ATAC haplotype, containing all wild-type alleles. Interestingly, the authors performed *in vitro* studies with luciferase constructs and showed that ATAC transfected cells had an increase of 50% in luciferase expression when compared to the variant haplotypes GATC and GATT (Duan *et al.*, 2011). This represents hard evidence that *ARG1* polymorphisms impact gene expression and suggests a mechanism by which those polymorphisms may alter bronchodilator responsiveness. 

The proposed mechanism of interaction between arginases and β_2_ adrenergic receptors involves NO and cGMP pathway. The ATAC haplotype would lead to increased expression of Arginase 1 (Duan et al., 2011), leading to lower availability of the substrate for NO synthesis. Consequently, this would lead to an increase in smooth muscle tonus in airways. When treated with bronchodilators, ATAC haplotype carriers would respond better to therapy, which is consistent with the concept of sensitization of the NO pathway (Cashen et al., 2002; Pereira et al., 2021). The idea is that when the NO-cGMP pathway is unstimulated, it would in turn increase its sensibility, since NO is needed tonically, even in small amounts. In this situation, stimuli that increase NO production and the machinery that respond to NO would respond in an increased intensity after an acute pharmacological stimulus. The opposite phenomenon also occurs when NO-cGMP is overstimulated, where it will reduce tissue responsiveness to chronic pharmacological stimulation (Cashen *et al.*, 2002; Pereira *et al.*, 2021). An example of this is the evidence that genetically engineered animals with eNOS knockout in aorta show an increased vasorelaxant effect in response to NO donors than wild type animals (Hussain et al., 1999), presumably by a sensitization of the NO-cGMP pathway. Up to date the evidence shown here is not used yet to tailor a personalized therapy for asthma. 

### Genetic influence of ARG1 and ARG2 in drug response in other diseases

Other groups explored the association of *ARG1* and *ARG2* polymorphisms with drug response in other contexts. Our group assessed the association between arginase 1 and 2 levels, activity and genetic polymorphisms in its genes with the responsiveness to the therapy of erectile dysfunction with a phosphodiesterase 5 (PDE5) inhibitor, Sildenafil (Lacchini et al., 2018). This drug acts downstream of NO signaling, enhancing the life span of the second messenger cGMP produced by soluble guanylate cyclase (which in turn is activated by NO). This is achieved by inhibiting PDE5, which is the main enzyme responsible for cGMP metabolization in cavernosal tissue. This allows for weaker stimuli to elicit an accumulation of cGMP with enough concentration to relax smooth muscle and initiate the erection process. Interestingly, it was shown that poor responders to Sildenafil that underwent prostate cancer surgery (more related to nerve damage) had an increased arginase activity in plasma, while poor responders of Sildenafil that were classified as clinical erectile dysfunction (more related to vascular dysfunction) showed an upregulation of Arginase 1 in plasma. Genotypes and haplotypes were assessed, including SNPs rs2781659 (A>G), rs2781667(C>T), rs2246012 (T>A) e rs17599586 (C>T) of *ARG1* and rs3742879 (A>G) e rs10483801 (C>A) of *ARG2.* While no associations were found of the SNPs and haplotypes with Sildenafil responsiveness, we found that variant genotypes CT of rs2781667(C>T), AG of rs2781659(A>G) and CT+TT of rs17599586(C>T), as well as GTTT haplotype of *ARG1*, were associated with a reduced arginase activity on plasma in clinical erectile dysfunction group. 

Another study focused on the same *ARG1* and *ARG2* SNPs, in the treatment with propofol (Oliveira-Paula et al., 2021). This drug is used as general anesthetic, and while it has a very short half-life, and patients recover conscience fast, this drug elicits an important blood pressure drop, which has many mechanisms, one of them involves the acute activation of NOS and NO production. Interestingly, this effect is sufficiently fast and intense to overcome the baroreflex, and blood pressure drops markedly. This is a special context in pharmacogenetics: since all counteracting mechanisms are exhausted and the cumulative response is fast, the subtle genetic effect on this phenotype may be more easy to observe, unraveling mild genetic effects that usually could be counteracted by physiological mechanisms. Indeed, it was shown in this study that *ARG2* rs3742879 (A>G) AG+GG carriers had a reduced mean arterial pressure drop and a reduced increase in plasma nitrite 5 minutes after propofol anesthesia, when compared to AA carriers. On the other hand, CA carriers of the *ARG2* rs10483801 (C>A), had a more intense mean blood pressure drop than CC wild-type carriers. At the same time, carriers of at least one variant allele of *ARG2* rs10483801 (C>A) showed increased levels of plasma nitrite, which indicates a more intense NO production (Oliveira-Paula *et al.*, 2021). While functional data are not available for these *ARG2* polymorphisms, results suggest that alleles that lead to an increased function of *ARG2* (which is highly expressed in endothelium) may lead to a reduction of L-Arginine availability in a compartmentalized fashion. This, in turn would lead to a diminished production of NO because of substrate exhaustion, leading thus to a reduced blood pressure drop following propofol stimuli. This is supported by animal model evidence. It was shown that the treatment with Simvastatin, L-citruline and arginase inhibitors were able to reduce vascular damage induced by Arginase 1 in animals (Romero et al., 2008). Besides, arginase inhibition was also able to reduce insulin resistance and prevent hypertension installment in animals (Peyton et al., 2018). Moreover, it was shown that coronary arterioles from diabetic patients would have their vasodilatory response to acetylcholine restored if a pretreatment with L-Arginine or arginases inhibitors was given (Beleznai et al., 2011). While very interesting, to date no clinical study assessed whether *ARG1* and *ARG2* SNPs would associate with diabetes end-organ damage, which is decurrent mostly by low oxygenation and oxidative stress induced by hypercontractility of peripheral vessels. However, it is important emphasize that in only three of seven articles revised there is a correlation between SNPs in *ARG1* and response to drugs, so caution is needed when interpreting this information.

### Endogenous inhibitors of eNOS

Asymmetrical dimethylarginine (ADMA), symetrical dimethylarginine (SDMA) and Monomethylarginine (L-NMMA) are methylated forms of L-Arginine. ADMA and L-NMMA are considered as endogenous inhibitors of eNOS, since they directly reduce activity of eNOS, iNOS and nNOS, while SDMA acts only indirectly (Bouras et al., 2013; Rochette et al., 2013).

Several studies linked ADMA to cardiovascular diseases (Bouras et al., 2013) and insulin resistance (Perticone et al., 2010). In disease states when ADMA is elevated, eNOS activity may be reduced by 30 to 70%, depending on the disease (Cardounel et al., 2007).

On the other hand, SDMA acts inhibiting a specific channel for L-Arginine, reducing its inflow into the cellular compartment (Rochette et al., 2013). Therefore, while plasma levels of L-Arginine may be within normality, endothelial cells have a reduced availability of L-Arginine and thus a reduced production of NO. The different forms of methylated arginine are metabolized by a mixed action of renal excretion and metabolism (Kielstein et al., 2006). In renal insufficiency, methylarginines excretion is reduced and both ADMA and SDMA accumulate in plasma. This represents a risk as increased levels of ADMA are associated with risk to develop renal diseases (Kielstein *et al.*, 2006), as well as cardiovascular diseases (Emrich et al., 2018).

Few enzymes have the ability to metabolize methylated forms of L-Arginine: dimethylarginine dimethylaminohydrolases types 1 and 2 (DDAH1 and DDAH2) and Alanine-Glyoxylate aminotransferase type 2 (AGXT2) (Valkonen et al., 2005) (Schepers et al., 2014)

DDAH1 and DDAH2

DDAH1 and DDAH2 are responsible for metabolizing ADMA systemically. Both isoforms are widely expressed throughout different organs and tissues, however the localization differs between the two proteins (Leiper et al., 1999). DDAH1 is mainly expressed in liver, kidneys and tissues that express nNOS (Leiper *et al.*, 1999; Mishima et al., 2004). On the other hand, DDAH2 is expressed by vascular endothelium (which also expresses eNOS) and immune cells (that express iNOS) (Tran et al., 2000). DDAH1 is encoded by the *DDAH1* gene, located in the short arm of chromosome 1, region 22, while DDAH2 is encoded by *DDAH2* gene, located in the short arm of chromosome 6, region 21.3 (Tran *et al.*, 2000).

Genetic studies showed that polymorphisms in *DDAH1* and *DDAH2* are associated with changes in ADMA levels, and when these are elevated, there is an increased risk to develop cardiovascular diseases (Leineweber et al., 2017) (Table 3). Despite the fact that there is in the literature evidence of ADMA levels associating with altered responsiveness to statins and hypoglycemic drugs (Maas, 2005), the genetic influence involving *DDAH1* and *DDAH2* polymorphisms has not been explored yet.

**Table 3 -:** Summary of studies that evaluated polymorphisms in response to drug for DDAH1 and DDAH2.

Gene	Polymorphism	Study Population	Disease or Procedure	Drug	Main Findings	Reference
*DDAH1*	rs1554597 (T>C) rs18582 (G>A)	Brazilian	Erectile dysfunction	Sildenafil	No significant associations	(Azevedo et al., 2017)
	rs1241321(A>G)	Chinese	Type 2 diabetes	Anti-platelet drug Statins ACE-Inhibitor/ARB Calcium channel blocker Hypoglycemic treatments	No significant difference between groups	(Lu et al., 2011)
*DDAH2*	rs805304 (C>A) rs805305 (C>G)	Brazilian	Erectile dysfunction	Sildenafil	AA and GG genotypes of rs805304 and rs805305 respectively were associated with a better response to treatment	(Azevedo et al., 2017)

**Figure 3 -:**
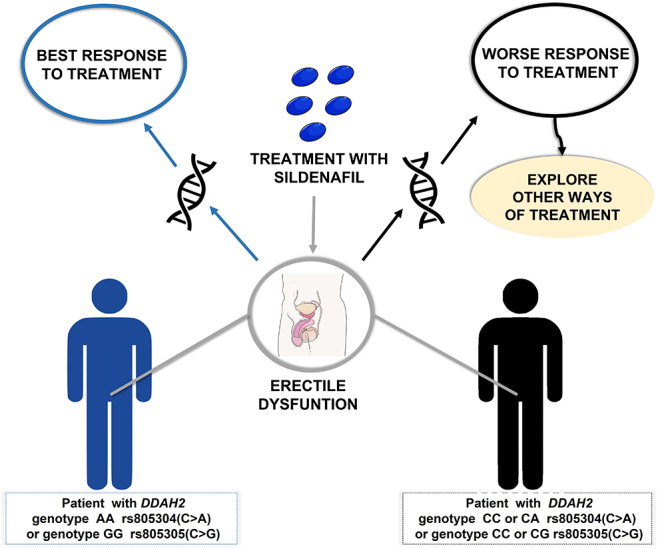
Possible future application of pharmacogenetic studies in clinical practice. Schematic figure, based on the results of Azevedo et al. (2017). *DDAH2,* Dimethylarginine dimethylaminohydrolase 2.

Interestingly, the reduced expression or activity of DDAH induces endothelial dysfunction (Leiper et al., 2007), and animal models showed that this, in turn, may lead to disease such as erectile dysfunction (Masuda et al., 2002; Park et al., 2009). Azevedo and coworkers assessed whether genetic polymorphisms of *DDAH1* (rs1554597 (T>C) and rs18582 (G>A)) and *DDAH2* (rs805304 (C>A) and rs805305 (C>G)) were associated with two types of erectile dysfunction (ED), Clinical ED and postprostatectomy ED (Azevedo *et al.*, 2017). Interestingly, rs18582 (G>A) A allele carriers had reduced ADMA levels, while the variant CC genotype carriers for rs1554597 (T>C), also showed reduced plasma levels of ADMA, both on clinical ED group (Azevedo *et al.*, 2017) (Figure 3). When considering inclusion/exclusion criteria, Clinical ED is a phenotype that is enriched by vasculogenic ED, while the postoperative ED group is enriched by nerurogenic ED. Interestingly, in postprostatectomy patients, *DDAH2* SNPs DDAH2 rs805304 (C>A) and rs805305 (C>G) that were not associated with changes in plasma levels of ADMA, associated with Sildenafil responsiveness. Interestingly, a Case-Control study regarding only Clinical ED, showed that *DDAH1* haplotypes including rs1554597 (T>C) and rs18582 (G>A) were associated with changes in ADMA levels of Clinical ED patients, where TG carriers shown increased levels, while CA carriers shown reduced levels on ADMA in plasma (Brites-Anselmi et al., 2019). On the other hand, *DDAH2* haplotypes were not associated with ADMA plasma levels, however did associate with plasma nitrite levels, whereas CC carriers had reduced nitrite levels and AG carriers shown increased nitrite levels in plasma (Brites-Anselmi *et al.*, 2019). Taken together, these data suggest that both enzymes have different roles, DDAH1, mainly expressed in liver, being more responsible for ADMA clearance, while DDAH2 may be compartmentalized within endothelial cells, where while it may not affect plasma ADMA levels, it may impact nitric oxide synthesis, because it affects ADMA levels in the compartment where NO is synthesized. This could also help to explain *DDAH2* SNPs associated with Sildenafil responsiveness. While it was already shown that DDAH genetic variability could impact ADMA plasma levels in other clinical settings (Leineweber et al., 2017), DDAH1 and 2 specific functions are yet to be tested in animal models to better understand the different physiological roles of both enzymes. 

## Vascular Endothelial Growth Factor

The Vascular Endothelial Growth Factor (VEGF) actions impact directly eNOS expression and NO bioavailability (Figure 1) (Yang et al., 2012). Interestingly, NO also regulates VEGF expression, therefore it is a reciprocal relationship (Lacchini et al., 2013). VEGF is encoded by a homonymous gene, located at chromosome 6, position p21. *VEGF* polymorphisms have been extensively studied in cancer in order to predict anticancer therapy outcome. In that setting VEGF signaling is crucial for tumor angiogenesis and growth, and therapies that target VEGF have the objective to limit blood flow to the tumor tissue. It was shown that colorectal cancer patients that were carriers of the TT genotype of the rs3025039 (located in the promoter, position -936) had better survival after treatment with Bevacizumab than their counterparts (Ulivi et al., 2015) (Table 4). This drug is a monoclonal antibody against VEGF that is used as an adjuvant therapy in certain types of cancer. Other polymorphisms in the same gene (rs699947, rs833061, rs2010963 e rs1570360) analyzed separately or in haplotype blocks did not associate with Bevacizumab responsiveness (Ulivi *et al.*, 2015). Another study focusing on colorectal metastatic cancer showed an association of the -1498 C>T polymorphism with Bevacizumab responsiveness: TT carriers had increased progression-free survival after treatment when compared to CC carriers (Loupakis et al., 2011). Another interesting anticancer drug that targets VEGF is Sunitinib (Eechoute et al., 2012), which is a tyrosine kinase inhibitor with multiple targets, that acts on VEGF signaling by inhibiting VEGF receptors and resulting in less angiogenesis, less tumor growth and reduced metastasis (Ferrara et al., 2003). Because of the impact of VEGF on NO and its role in blood pressure control, one of the main adverse effects of Sunitinib is blood pressure increase. A study assessed the association of blood pressure increases and survival after sunitinib use in metastatic renal cell cancer patients (Eechoute *et al.*, 2012). This retrospective study showed that carriers of the ACG haplotype (composed by rs699947, rs833061 e rs2010963 in *VEGF* gene) had increased systolic and mean arterial pressure after Sunitinib treatment. However, the same haplotype was also associated with increased survival, with a median survival time increase of 7.2 months (Eechoute *et al.*, 2012). These results are consistent with the idea that ACG haplotype carriers had an increased inhibition of VEGF, and that this increased blood pressure, also reduced angiogenesis at the tumor site increasing survivability. Equivalent observations were reported, associating higher blood pressure to good responsiveness to Sunitinib (Gallagher et al., 2011; Rini et al., 2011; Szmit et al., 2012). 

**Table 4 -:** Summary of studies that evaluated the association of polymorphisms in *VEGF* with response to drugs.

Polymorphism	Study Population	Disease	Drug	Main Findings	Reference
rs699947 (2578C>A) rs1570360 (1154G>A) rs2010963 (634G>C)	Brazilian	Erectile Dysfunction	Sildenafil	Genotype AA and CA carriers of rs699947 had worse response to Sildenafil in Clinical Erectile Dysfunction (CED) group. Carriers of AA genotype for rs1570360 showed worse responses in postoperative (PED) and CED. AAG haplotype carriers had increased risk to worse responses to Sildenafil.	(Lacchini et al., 2013)
rs699947 (−2578A>C) rs833061 (−460C>T) rs2010963 (405C>G)	Netherlands	Metastatic renal cell cancer (mRCC)	Sunitinib	ACG haplotype carriers showed increased risk to increase in systolic and mean arterial pressure after Sunitinib treatment.	(Eechoute et al., 2012)
rs699947 (2578C>A) rs1570360 (1154G>A) rs2010963 (-634G>C)	Brazilian	Hypertension	Enalapril (Angiotensin-converting Enzyme Inhibitor)	rs699947 AA and CA genotype carriers and AGG haplotype carriers had a more intense blood pressure drop after Enalapril treatment. Carriers of rs699947 CC genotype and the CGG haplotype responded less to Enalapril treatment	(Oliveira-Paula et al., 2015)
−2578C>A −1498C>T −1154G>A −634C>G +936C>T	Italian	Metastatic colorectal cancer	Bevacizumab	Carriers T/T genotype of 936 polymorphism had shorter median progression-free survival	(Ulivi et al., 2015)
rs3025039 (936 C>T)	Chineses Han	Coronary Artery Disease	Drug-eluting stent (DES)	No significant differences	(Zeng et al., 2017)
-2578 C>A -1498 C/T, -405 C/G -936 C/T	Italian	metastatic colorectal cancer	Bevacizumab	Carriers of the TT genotype for 1498 C/T had a reduced progression-free survival than their counterparts	(Loupakis et al., 2011)

As the evidence shows, *VEGF* polymorphisms may have an important role in blood pressure regulation. Interestingly, some anti-hypertensive drugs increase VEGF within their mechanism of action, such as Angiotensin Converting Enzyme Inhibitors (ACEi), and this may be important for the clinical response observed for these anti-hypertensive drugs (Li et al., 2008; Yazawa et al., 2011). It was shown that the blood pressure response to Enalapril, which is an ACEi, associated with polymorphisms in *VEGF* (Oliveira-Paula et al., 2015). Carriers of AA and CA genotypes for rs699947 and carriers of the AGG haplotype (composed by rs699947, rs1570360 e rs2010963) had more intense blood pressure drops after Enalapril treatment, when compared to CC genotype of rs699947 and CGG haplotype (Oliveira-Paula *et al.*, 2015). This suggests that *VEGF* polymorphisms may affect blood pressure control in a large portion of hypertensive patients, since this drug is frequently used. In clinical erectile dysfunction, it was shown that rs699947 AA and CA genotype carriers and AGG haplotype carriers responded worse to Sildenafil (Lacchini et al., 2013). While the association with enalapril seems contrasting with the association with Sildenafil at a first glance, the authors discuss that since there is an important effect of tachyphylaxis in NO pathway, it could be possible that haplotypes and genotypes associated with chronic higher production of NO could in turn lead to a reduced cGMP accumulation following PDE-5 inhibition (Oliveira-Paula *et al.*, 2015). Since Clinical ED has endothelial dysfunction as the limiting step in vasodilation, this may be more visible in this condition. Postoperative ED, on the other hand had an association of AA genotype of rs1570360 with worse Sildenafil responsiveness (Lacchini *et al.*, 2013). Both polymorphisms implicated in this study are functional, and the variant allele shows reduced expression of VEGF (Shahbazi et al., 2002; Lambrechts et al., 2003). Altogether these results provide good evidence that *VEGF* polymorphisms may impact drug response, especially when considering vasodilation and angiogenesis mediated by this molecule.

### Other pathways less explored

AGXT2

The AGXT2 enzyme is the main enzyme responsible for symmetrical dimethylarginine (SDMA) metabolism, and also responsible for around 16% of the ADMA intracellular metabolism (Schepers et al., 2014). SDMA, as discussed before, is a molecule involved in inhibiting NO synthesis, especially by inhibiting L-Arginine channels that are essential for providing adequate substrate for NO synthesis in endothelial cells. AGXT2 is encoded by a homonymous gene, located at 5p13.2 (Rodionov et al., 2014). Interestingly, polymorphisms in this gene were associated with increased risk to cardiovascular diseases, including associations with intermediate phenotypes, such as reduced enzyme activity, increased ADMA and SDMA and reduced NO biomarkers (Hu et al., 2016; Yoshino et al., 2021). There are functional SNPs in this gene, such as the rs37369 (A>G), that besides associated with changes in renal and liver clearance of ADMA, is also associated with a marginal increase in survival time in heart failure patients: A carriers survived more than GG carriers (Hu *et al.*, 2016; Yoo et al., 2021). While these results show an exciting perspective in drug response prediction, there are no studies in the literature exploring this association with drug response. 

Soluble Guanylate Cyclase

The Soluble Guanylate Cyclase (sGC) enzyme acts as an intracellular sensor of NO (Arnold et al., 1977). When in the presence of NO, sGC converts guanosine triphosphate (GTP) in cyclic guanosine monophosphate (cGMP) (Derbyshire and Marletta, 2012). sGC is the mediator for NO signaling, that begins with NO synthesis by NOS (Derbyshire and Marletta, 2012). Because of the well-established role of NO in the cardiovascular system, sGC is also mainly associated with cardiovascular diseases (Gheorghiade et al., 2013). sGC is a heterodimeric enzyme, consisting of alpha and beta subunits, which, in turn, have two main isoforms each: α_1_, α_2_, β_1_, β_2_ (Russwurm et al., 1998; Mergia et al., 2003). A large study including 2012 hypertensives and 2210 healthy controls assessed the association of hypertension with polymorphisms in chromosome 4, in a region comprising the genes *GUCY1A3* to *GUCY1B3* (which encode sGCα_1_ and sGCβ_1_, respectively). This included six SNP: rs3806777, rs3806782, rs3796576 and rs7698460 at *GUCY1A3*, as well as rs2229202 and rs1459853 at *GUCY1B3* (Chen et al., 2019). Patients that carry the AA genotype of rs1459853 (G>A) had increased risk for hypertension than GG and GA carriers. When analyzing by age, it was shown that in adolescents, the TT genotype for rs2229202 (C>T) was associated with increased risk to hypertension and prehypertension when compared to CT and CC. Interestingly, no study assessed the association of these SNPs with drug response, although several drugs act through this gene product, such as NO donor vasodilators, for instance.

## Conclusion and future perspectives

Despite the fact that there are few articles in the literature associating genetic polymorphisms in genes of the NO pathway (other than *NOS3*) with drug response, there is consistent evidence showing the importance of these genetic markers possibly affecting drug response and treatment outcome. It is interesting that the association of these polymorphisms with disease risk is much more explored and several markers that alter disease risk also were associated with drug response. This shows that there is a large potential in studying pharmacogenetics, especially when considering candidate pathways instead of candidate genes. This may also prove additional value when considering that most researchers nowadays prefer to study genome wide data. Pathway analysis may be complemented with biochemical biomarkers closely related with these pathways, which could also provide mechanistic insights on how these markers may affect these complex phenotypes. Large scale explorative analysis could be financially prohibitive or be experimentally limited if one considers genome wide data in parallel with proteomic and metabolomic data. Some biochemical biomarkers require specific pre-assay handling and/or preparation to be properly assessed, which may be overlooked in large scale analyses. Therefore, pathway genetic association studies have their own value and may be important to establish clinically important associations. While it is very clear that common SNPs may lead to subtle effects, not determining phenotypes, it is very interesting to see that results reported here are reproduced between different clinical settings (cancer, cardiovascular diseases, urological diseases) that are affected by NO availability. Albeit the large potential, there is still much to be studied in this field to provide reliable, sensible, precise and clinically relevant genetic markers with capabilities to personalize the drug regimen for each genetically unique patient.
